# Gynecological hysterectomy in Northern Tanzania: a cross- sectional study on the outcomes and correlation between clinical and histological diagnoses

**DOI:** 10.1186/s12905-020-00985-9

**Published:** 2020-06-12

**Authors:** Daniel Michael, Alex Mremi, Patricia Swai, Benjamin C. Shayo, Bariki Mchome

**Affiliations:** 1 Department of Obstetrics and Gynecology, Kilimanjaro Christian Medical University College, Box 2240, Moshi, Tanzania; 2 Department of Pathology, Kilimanjaro Christian Medical Center, Box 3010, Moshi, Tanzania; 3 Department of Obstetrics and Gynecology, Kilimanjaro Christian Medical Centre, Box 3010, Moshi, Tanzania

**Keywords:** Outcome, Hysterectomy, Histology,Tanzania

## Abstract

**Background:**

Hysterectomy is one of the most common gynaecological procedures performed worldwide. The magnitude of the complications related to hysterectomy and their risk factors are bound to differ based on locations, availability of resources and level of surgical training. Documented complications rates and their correlates are reported from high income countries while data from low- and middle-income countries including Tanzania is scare.

**Methods:**

This was a hospital based cross-sectional study conducted at a tertiary facility in northern Tanzania where 178 women who underwent elective gynecological hysterectomies in the department of obstetrics and gynecology within the study period were enrolled. Logistic regression was performed to determine the association between risk factors and occurrence of surgical complication where *p*-value of < 0.05 was considered statistically significant. The degree of correlation between pre-operative clinical and histological diagnosis was determined by kappa correlation test.

**Results:**

A total of 75 (42%) of women had surgical complications within 10 days of surgery. Blood transfusion and intra-operative bleeding were the most common complications observed in 34 (19.1%) and 17 (9.6%) women respectively. Independent risk factors for complications included obesity (OR 3.9; 95% CI 1.44–10.46), previous abdominal operations (OR 8.44; 95% CI 2.52–28.26) and longer duration of operation (> 2 h) (OR 5.02; 95% CI 2.18–11.5). Both uterine fibroid and adenomyosis had good correlation of clinical and histological diagnosis (*p*-value < 0.001).

**Conclusion:**

Bleeding and blood transfusion were the most common complications observed in this study. Obesity, previous abdominal operation and prolonged duration of operation were the most significant risk factors for the complications. Local tailored interventions to reduce surgical complications of hysterectomy are thus pivotal. Clinicians in this locality should have resources at their disposal to enhance definitive diagnosis attainment before surgical interventions.

## Background

Incidence of gynecological hysterectomy varies between countries. USA and Germany have reported 600,000 and 34,872 hysterectomies annually respectively [1, 2]. Kano hospital in Nigeria reported a hysterectomy rate of 5.1% [3], and in Korle Bu teaching hospital in Ghana it was found to be 7.8% [4].

Indications for gynecological hysterectomy include uterine fibroid, adenomyosis, intraepithelial neoplasm (CIN), prophylaxis against uterine cancer, endometrial adenocarcinoma e.t.c [5–7]. As with all kinds of surgery, hysterectomy can have complications as well. Some of the reported complications include hemorrhage, urinary tract injury, surgical site wound infection, fever, blood transfusions, re-operation, organ lesions and re hospitalizations [8, 9].

Factors associated with surgical complications following gynecological hysterectomy have also been studied and do vary. High body mass index (BMI), multi parity and previous abdominal scars have been associated with significant complications [10].

Histological examination is mandatory of uterine specimen after hysterectomy. Studies done in India, Pakistan and Rwanda found leiomyoma to be the most common histological finding [11–13]. Correlation between clinical and histological pattern is important in the general management of the patients. Studies done in India, Bangladesh and Nigeria found the correlation between pre-operative clinical and histological diagnoses were 74, 77 and 95.6% respectively [11, 14, 15].

No published data to account for the complication rates and the risk factors of hysterectomy among women in Tanzania. The primary intent of the current investigation was to determine surgical outcomes, associated risk factors, histological pattern of uterine specimens, and the correlation between pre-operative clinical and histological diagnoses at Kilimanjaro Christian Medical Centre (KCMC), a tertiary referral and zonal hospital in Northern Tanzania.

## Methods

### Study design

This was a hospital based cross-sectional study, designed to obtain reflection of patients who underwent elective gynecological hysterectomy at KCMC hospital, in northern Tanzania. The study was carried out from July 2018 to May 2019.

### Study setting

This study was conducted at KCMC referral and tertiary hospital located in Kilimanjaro region in northern Tanzania. According to 2012 national census, Kilimanjaro region has estimated population of 1,640,087 people. KCMC is a referral hospital for over 15 million people in northern Tanzania and the gynecology department conducts an approximate 500 gynecological operations (elective and emergency) in a year.

### Study participants

All women who were scheduled for elective gynecological hysterectomy were eligible to participate in the study. A simple review of elective gynecological hysterectomies that were performed in previous years was done and a minimum sample size of 163 was expected during the study period.

### Data collection

Questionnaires were handed out to collect information from all the women who met inclusion criteria.

A day before the elective operation, research assistant approached the participants and requested their participation to the study. Written informed consent was sought after risk and benefit of participating into the study were described. Once the consent forms were signed, face to face interview using a structured questionnaire was conducted to obtain important baseline characteristics of the participants. A follow up was done after surgery where relevant information was abstracted from operation and anesthesia notes.

Patients were followed up post operative in the ward through ward round notes (every day) to follow their progress until discharge. After being discharged, they were followed via telephone interviews (every two days), and on the 10th day post operation patient were discharged from the study. Patients who were found to have complications post discharge were all asked to be re-admitted for appropriate management. Two weeks after operation, histology reports were traced from pathology department.

### Data analysis

Collected data was checked for duplicates, missing information and validity using Microsoft excel. Thereafter, data was transferred to STATA (Version 13.0) for analysis. Descriptive statistics was used to summarize the study variables. The numerical data were summarized using mean and standard deviation while categorical data was summarized using frequency and percentage presented in tables and figures.

The independent variables like age in years, parity, hypertension, diabetes mellitus, hemoglobin (Hb) level before operation, hysterectomy type, duration of operation in hours, clinical diagnoses- fibroid, endometrial hyperplasia, pre-malignant lesion of the cervix, BMI (kg/m^2^), previous abdominal operation were run using bivariate logistic regression analysis for the crude Odds ratio to identify their association with adverse surgical outcome of gynecological hysterectomy at 95% confidence level. Variables that indicated significant influence of adverse surgical outcome of gynecological hysterectomy like BMI (kg/m^2^), previous abdominal operation, and duration of operation in hours were then run into multivariate logistic regression analysis to control possible modifiable effects or confounders.

## Results

**Fig. 1:**
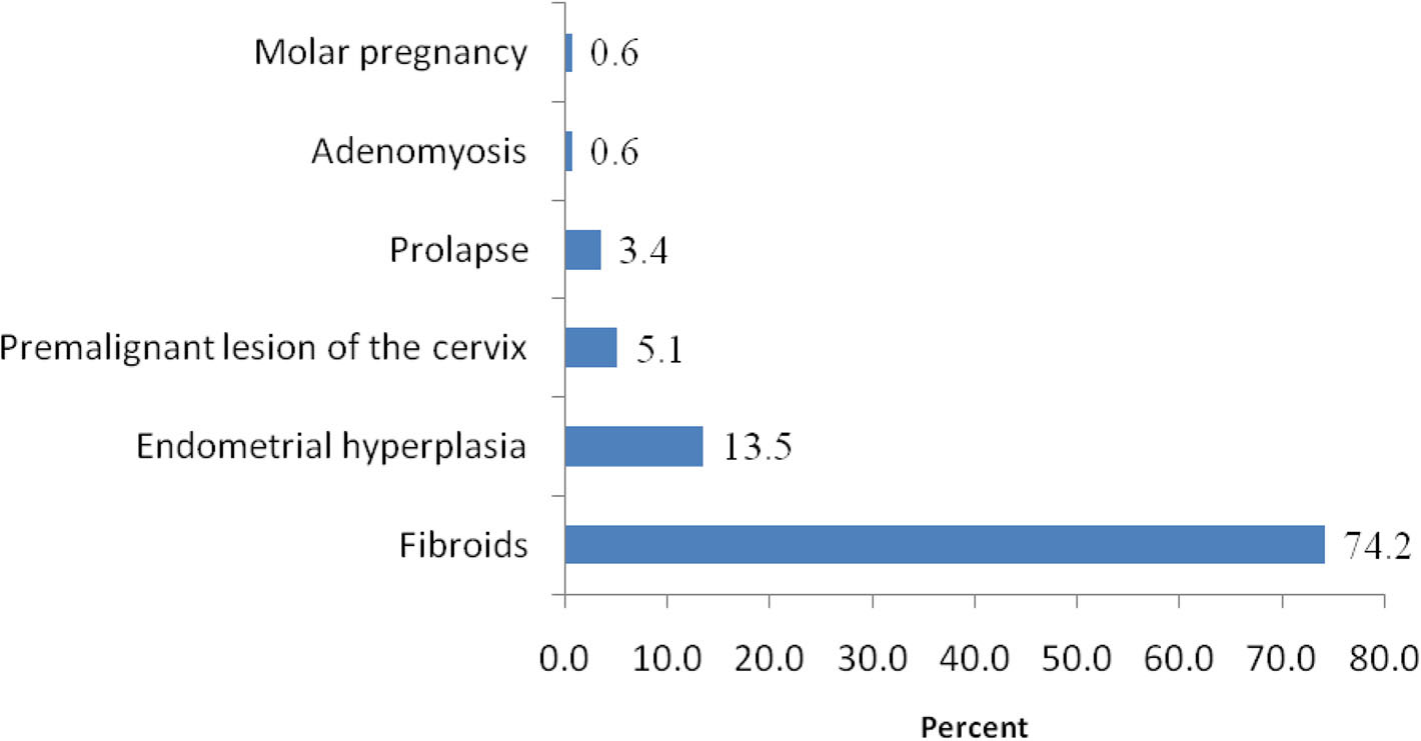
Indication for gynecological hysterectomy (*n* = 178)

During the study period, a total of 437 gynecological operations (both electives and emergency) were performed. Of these, 178(40.7%) were elective gynecological hysterectomy cases due to various indications, whereas, the rest included but not limited to myomectomies, salpingectomies, cystectomies, vaginal wall repairs, etc., were excluded (Fig. 1). The age distribution of the study participants ranged from 26 to 85 years with mean age of 48.8 ± 8.6 years; 15 (8.4%) of them were nulliparous with the rest being parous with mean parity of 3.3 ± 2. Majority of the study participants were predominantly overweight 80(44.9%), while only 24 (13.5%) of them had previous history of abdominal operation before hysterectomy. Abdominal hysterectomy was the most common type of hysterectomy observed in 168(94%) women (Table 1).

**Table 1:** Baseline characteristics of the Study Participants (*N* = 178)

Characteristics	Number	Percent
**Age, years**		
**Mean ± SD**	48.8 **±** 8.6	
26–44	57	32.0
45–64	112	62.9
65+	9	5.1
**Education level**		
Non-formal	34	19.1
Primary	61	34.3
Secondary	59	33.1
College/high level	24	13.5
**Marital status**		
Single	15	8.4
Cohabiting	6	3.4
Married	133	75.1
Divorced	10	5.6
Widowed	14	7.9
**Parity**		
**Mean ± SD**	3.3 **± 2**	
Nuliparous	15	8.4
1–2	43	24.2
3+	120	67.4
**BMI (kg/m**^**2**^**)**		
< 18.5	2	1.1
18.5–24.9	45	25.3
25.0–29.9	80	44.9
30+	51	28.7
**Hypertensive**		
No	144	80.9
Yes	34	19.1
**Diabetes**		
No	166	93.3
Yes	12	6.7
Others		
**Hb level before surgery(g/dl)**		
> 11	129	72.5
10.5–8.0	49	27.5
< 7.0	0	0
**Previous abdominal operation**		
No	154	86.5
Yes	24	13.5
**Hysterectomy approach**		
Abdominal	168	96%
Vaginal	10	4%

### Surgical complication of gynecological hysterectomy (*n* = 75)

The incidence of surgical complications as classified by Clavien and Dindo system was as follows: Degree I: 36(31.6%), Degree II: 65(57%), Grade III: 13(11.4%), Grade IV and Grade V: 0 (0%), Table 2. Intra operatively, 17(9.6%) of women had severe blood loss, 34(19.1%) had blood transfusion and 13(7.3%) sustained visceral injury (Table 2). Postoperatively, 44(24.7%) of cases had prolonged hospital stay (4 days or more), 18(10.1%)] of them had post-operative fever and 8(4.5%) had wound infection, (Table 2).

**Table 2:** Surgical complications of gynecological hysterectomy according to Clavien-Dindo classification

Complication	CLAVIEN-DINDO CLASSIFICATION			(GRADE)		
	I	II	III	IV	V	Percentage
Blood transfusion	0	48	0	0	0	27
Visceral Injury:	0	0	3	0	0	1.7
Ureteric injury	0	0	6	0	0	3.4
Urinary bladder injury Bowel injury	0	0	4	0	0	2.2
Post operative fever	18	0	0	0	0	10.1
Hemorrhage	0	17	0	0	0	9.6
Anemia	10	0	0	0	0	5.6
Wound infection	8	0	0	0	0	4.5
**Total**	**36 (31.6%)**	**65 (57.0%)**	**13 (11.4%)**	**0 (0.0%)**	**0 (0%)**	**100**

### The risk factors for surgical complications associated with gynecological hysterectomy

Table 3 shows the association between surgical complications and background characteristics. Obese women had 3 times higher odds of having surgical complications during gynecological hysterectomy than non-obese women. Likewise, women who had previous history of abdominal operation had 9 times higher odds of developing surgical complications during gynecological hysterectomy. Also, women whose duration of operation was more than 2 h had 4 times her odds of having surgical complications their counterparts.

**Table 3:** Multivariate logistic regression analysis for the adjusted odds ratio of hysterectomy related complications by the associated factors (*n* = 178)

Variables	Adjusted OR	95%CI	***P***-value
**BMI (kg/m**^**2**^**)**			
Normal:18.5–24.9	1.00		
Underweight: < 18.5	5.63	0.31–102.82	0.244
Overweight: 25.0–29.9	1.92	0.78–4.74	0.158
Obesity: ≥ 30	3.89	1.44–10.46	0.007
**Previous abdominal operation**			
No	1.00		
Yes	8.44	2.52–28.26	0.001
**Duration of operation in hrs**			
≤2	1.00		
> 2	5.02	2.18–11.58	< 0.0001

### Histological pattern of uterine specimens after gynecological hysterectomy cases (*n* = 168)

**Fig. 2:**
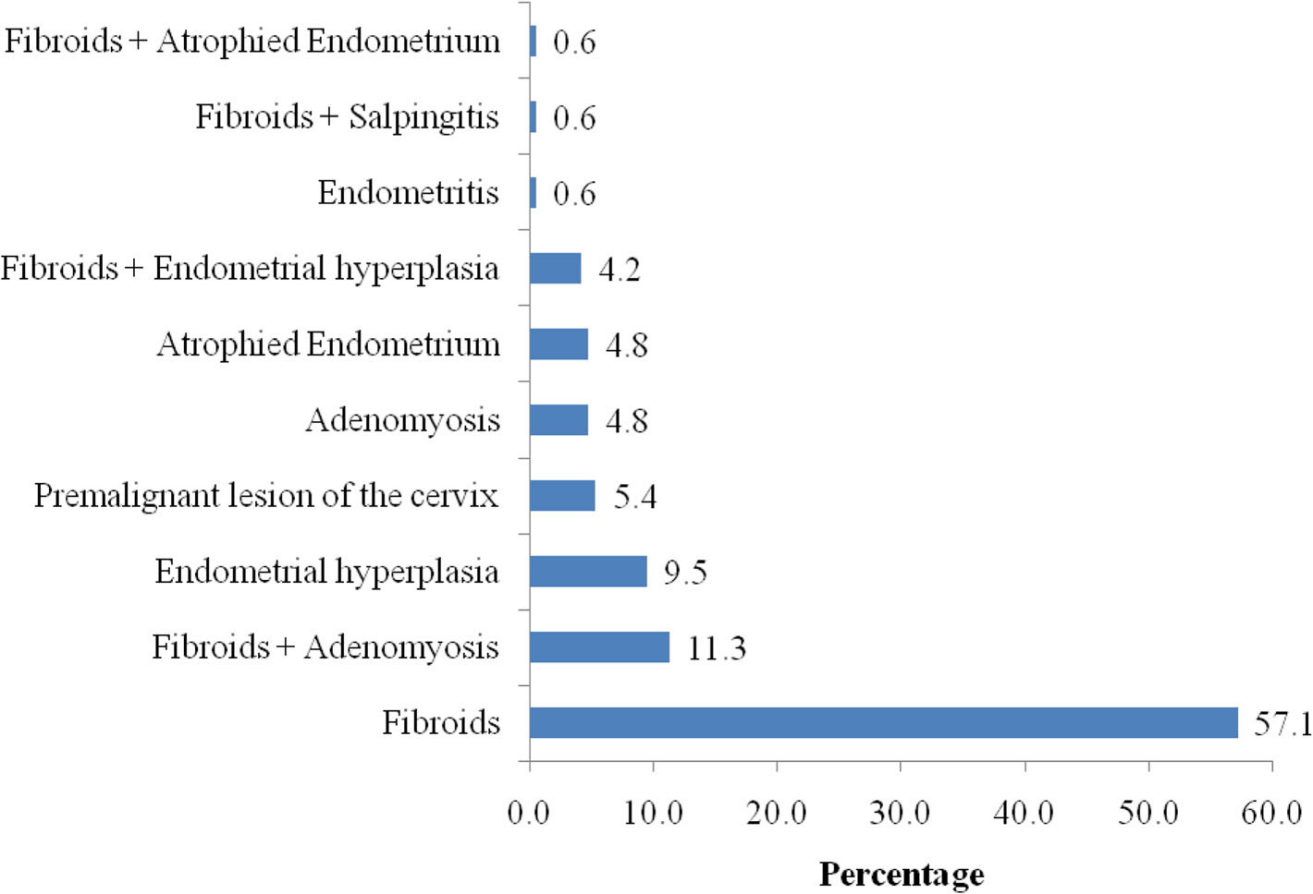
The histological pattern of uterine specimen submitted after gynecological hysterectomy (*n* = 165)

On histopathology finding of uterine specimen submitted after gynecological hysterectomy, a number of cases had more than one type of lesion, and each type of lesion was counted separately. The top most histological findings were uterine fibroid in 96 (57.1%) women, followed by uterine fibroid with adenomyosis in 19(11.3%) women, while the least observed was uterine fibroid with atrophied endometrium in 10(0.6%) women (Fig. 2).

### Correlation between pre-operative clinical diagnosis and histological diagnosis

**Fig. 3:**
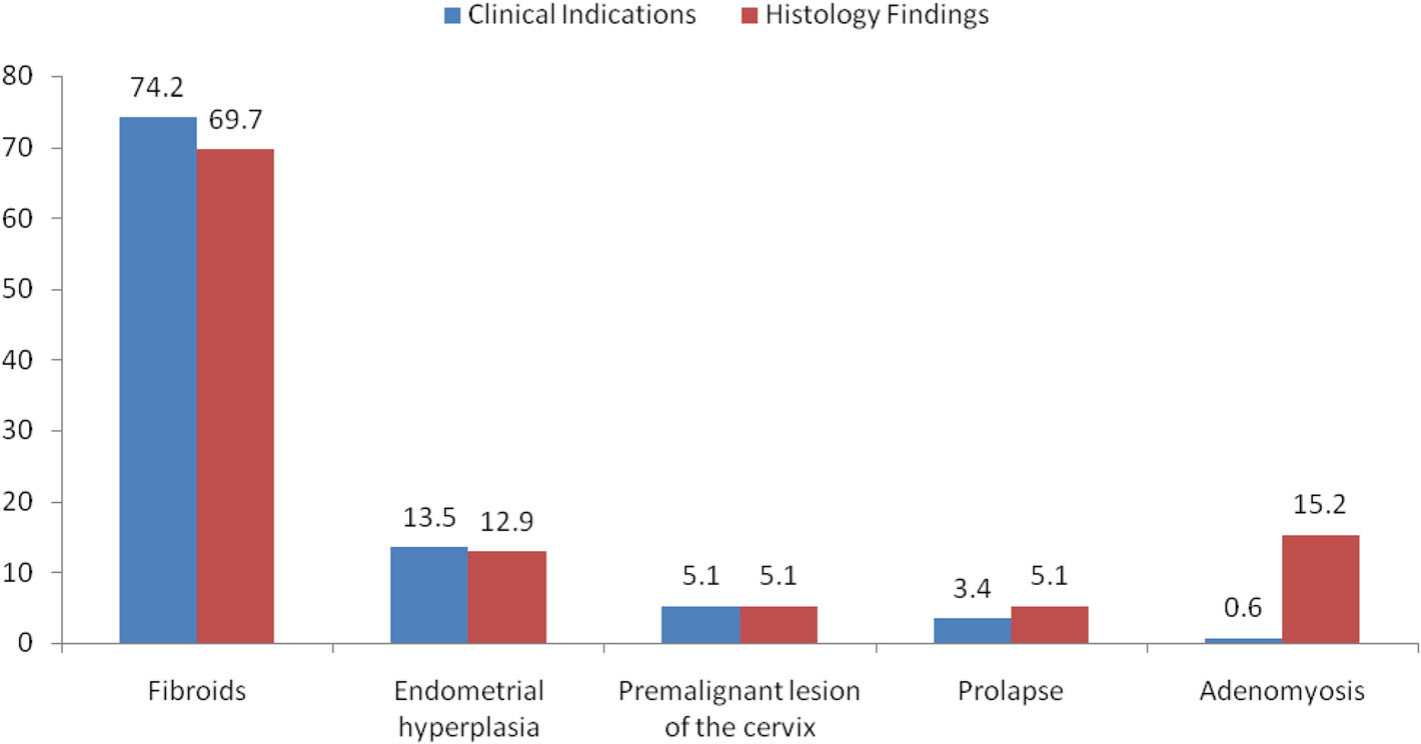
Correlation between pre-operative clinical diagnosis and histological diagnosis

Correlation between histological diagnosis and clinical diagnosis of uterine fibroid was 83%, the remainder (17%) of which consisted of some others incidental findings. Cases of pelvic organ prolapsed showed mainly atrophied endometrium on histology. Correlation between endometrial hyperplasia and premalignant lesion of cervix was more than 90% because their histology report is normally obtained before operation. Adenomyosis was mainly histological diagnosis (Fig. 3).

### Incidental findings of uterine specimen submitted for histopathology with the pre-operative clinical diagnosis of uterine fibroid (*n* = 123)

**Fig. 4:**
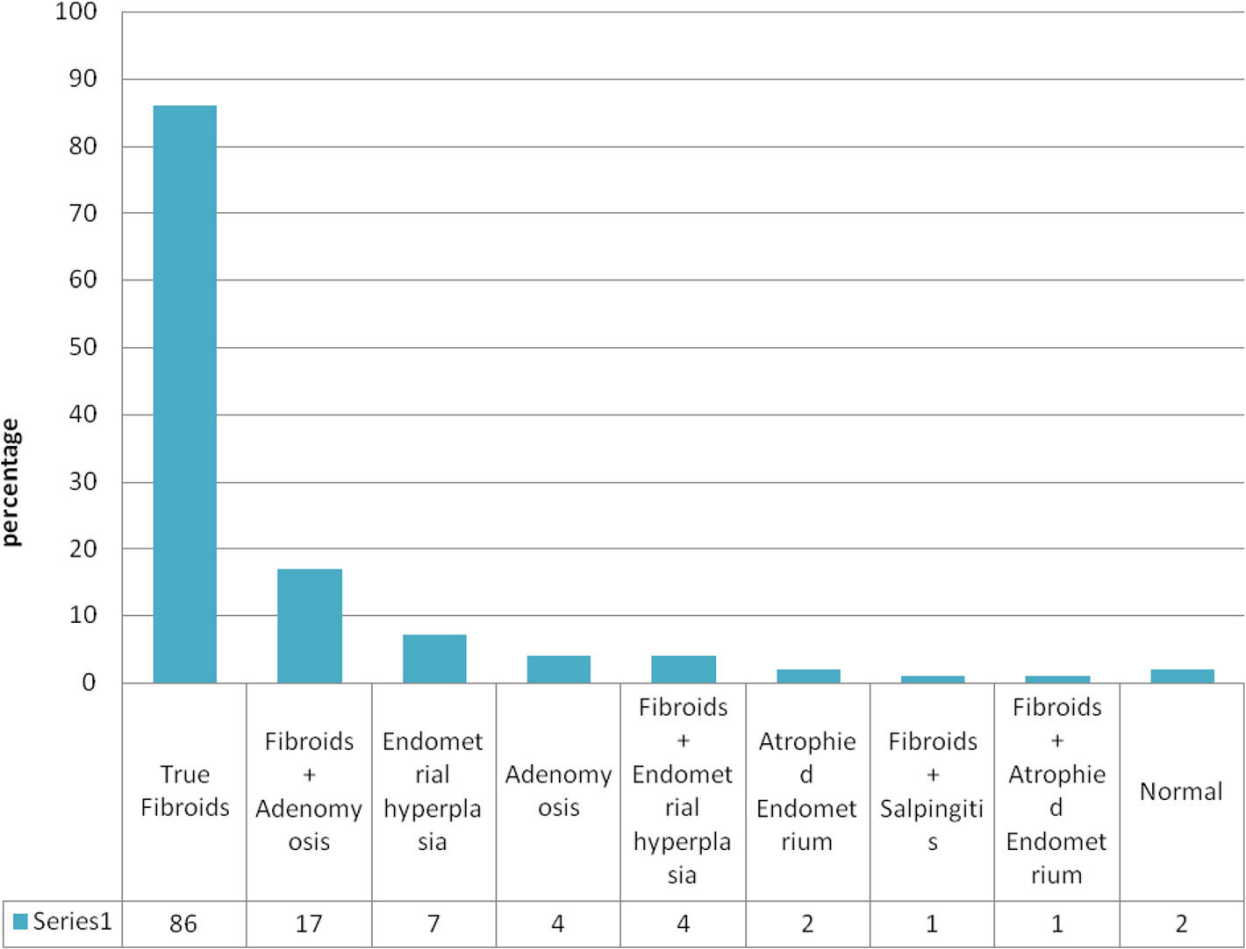
Incidental lesions found in hysterectomy specimens with pre-operative clinical diagnosis of uterine fibroid

Uterine fibroid was the most common clinical and histological findings, since the correlation of clinical and histological diagnosis was 83%, Fig. 4 below tried to look other histological findings which were seen in the uterine specimens for those patients with clinical diagnosis of uterine fibroids.

The degree of correlation between clinical and histological diagnosis has been shown in Table 4.

**Table 4:** Kappa Statistics for the Correlation between pre-operative clinical diagnosis and histological diagnoses

Clinical Indicators	Histological diagnosis correlated		
	Not correlated (%)	Correlated (%)	***P***-value
Fibroids	23 (17.4)	109 (82.6)	< 0.0001
Endometrial hyperplasia	14 (58.3)	10 (417)	< 0.0001
Premalignant lesion of the cervix	3 (33.3)	6 (66.7)	0.8310
Adenomyosis	8 (29.6)	19 (70.4)	< 0.0001

In this study, correlation between clinical diagnosis and histological findings of uterine fibroid, endometrial hyperplasia, uterine vaginal prolapsed, adenomyosis was found to be of statistically significant (Table 4).

## Discussion

In the current study, the commonest surgical complication was blood transfusion and intra operative bleeding. Obesity, previous abdominal surgery and longer duration of surgery were significantly associated with higher risk for surgical complications. The most common histological findings were uterine fibroid and the correlation between pre-operative clinical diagnoses and postoperative histological diagnosis was high at 83%.

In the current, study most common surgical complications observed were blood transfusion and prolonged hospital stays, this findings was similar with the findings which was observed in Nigeria and Ghana respectively [3, 4]. As abdominal hysterectomy was predominant route of hysterectomy in these studies, this can explain similarity. Furthermore, the study done in Ghana by Takyi et al. involved also peripartum hysterectomy which could be associated with high rates of bleeding and blood transfusions than the current study. In contrast to current study, different findings were observed in a study which was done in India [16], which reported high complications rates. The difference observed may be due to inclusions of only elective benign cases in this study.

In the present study the risk of developing complication was strongly associated with obesity and prolonged duration of operation. Similar findings was noted in a study which was done in Denmark by Osler and his colleagues [9]. The Danish study was specific at assessing effect of obesity on complications while our study into more risk factors other than weight for association with surgical complications. The studies done in India and Ghana respectively had different observation on the risk factors for surgical complications during gynecological hysterectomy [4, 17]. We were not able to make a comparison of surgical complications by route of hysterectomy. This is because our study had very small number of vaginal hysterectomies compared to abdominal hysterectomy thus it was not statistically meaningful.

In our study, uterine fibroid was by far the most common histological findings among uterine specimens submitted to pathology department following hysterectomy, correlating with findings from studies done in Pakistan and Rwanda [11, 13]. This similarity can be due to all patients involved in both studies were planned for hysterectomy and uterine fibroid is known as a worldwide leading indication of elective hysterectomy. Contrary to our findings, different observation was demonstrated in Sri Lanka and Yemen respectively [18]. The difference could be mainly attributed by our inclusion of only elective and benign cases.

In current study the correlation between pre-operative clinical diagnosis and histological findings was high (83%). This finding correlated with studies done elsewhere [19]. In a retrospective study done in India to assess the clinico-pathological correlation in a rural setting involving 368 hysterectomy specimens, authors reported correlation of 84.4% for benign conditions, [19]. In another retrospective descriptive study at two teaching hospitals in Rwanda, a total of 299 uterine specimens underwent histopathological assessment post hysterectomy; overall, 83% of the pre-operative clinical diagnoses were confirmed by histology [13].

Given the nature and logistical dynamics of the current study, findings and conclusion drawn from this study may not necessarily be representative of all complication rates and risks factors of hysterectomy in this region. A relatively limited sample size with few adverse events has been prohibitive in providing a more precise estimate of the magnitude of complication of hysterectomy. We additionally acknowledge complications provided in the current study may not have been further qualified to provide more descriptive information including types, and causative factor especially for post-operative fever and anemia. Furthermore, the nature of a cross-sectional design does not provide the opportunity to ascertain a temporal trend between exposure and outcome.

## Conclusion

This study has shown that 42% of patients who underwent gynecological hysterectomy had complications. Uterine fibroid was the leading indication of hysterectomy. Prolonged hospital stays was the most common complication observed, while obesity and prolonged duration of operation were significantly associated risk factors. Furthermore, uterine leiomyoma was the most common histological finding observed in gynecological uterine specimen submitted for histological review. The correlation between clinical and histological diagnosis was 83%.

## Data Availability

The datasets used and analyzed in the current study are available from the corresponding author upon special request.

## Abbreviations

BMI: Body mass index
CI: Confidence interval
CIN: Cervical intraepithelial neoplasm
CRERC: College Research Ethics and Review Committee
Hb: Hemoglobin
KCMC: Kilimanjaro Christian Medical Center
KCMUCo: Kilimanjaro Christian Medical University College
OR: Odds ratio
USA: United States of America

## References

[1] Lewin SN, Lu Y, Neugut AI, Dawn L (2014). NIH Public Access.

[2] Prütz F, Knopf H, Von Der Lippe E, Scheidt-Nave C, Starker A, Fuchs J (2013). Pr??valenz von Hysterektomien bei frauen im Alter von 18 bis 79 Jahren: Ergebnisse der Studie zur Gesundheit Erwachsener in Deutschland (DEGS1). Bundesgesundheitsblatt - Gesundheitsforsch - Gesundheitsschutz.

[3] Ahmed ZD, Taiwo N. Indications and Outcome of Gynaecological Hysterectomy at Aminu Kano Teaching Hospital, Kano : A 5-Year Review. Open J Obstet Gynecol. 2015;(May):298–304.

[4] Rabiu A, Habib R. Elective abdominal hysterectomy: Appraisal of indications and complications at Aminu Kano Teaching Hospital – An 8-year review. Trop J Obstet Gynaecol. 2017;34:224–8.

[5] Pandey D, Sehgal K, Saxena A, Hebbar S, Nambiar J, Bhat RG (2014). An audit of indications, complications, and justification of hysterectomies at a teaching hospital in India. Int J Reprod Med.

[6] Onyeabochukwu D, Duke-Onyeabo C, Onyegbule O, Amajuoyi C, Madu P (2014). A six year review of hysterectomy for benign gynaecological conditions at the Federal Medical Centre, Owerri. Int J Reprod Contracept Obstet Gynecol.

[7] Desai S, Campbell O, Sinha T, Mahal A, Cousens S. Incidence and determinants of hysterectomy in a low-income setting in Gujarat, India. Health Policy Plan. 2017(August 2016):68–78. 10.1093/heapol/czw099 PMC5886266 27497139

[8] Reddy MVN, Reddy MR. Comparison of total abdominal , vaginal and total laparoscopic hysterectomy. Int Surg J. 2016;3(4):2007–11. 10.18203/2349-2902.isj20163183.

[9] Osler M, Daugbjerg S, Frederiksen BL, Ottesen B. Body mass and risk of complications after hysterectomy on benign indications. J Reprod Epidemiol. 2011;26(6):1512–8. 10.1093/humrep/der060. 10.1093/humrep/der060 21467207

[10] Okafor CI. Original article A Review of Gynaecological Hysterectomies in a Private Specialist Hospital in Nigeria. Orient J Med. 2012;24:53–7.

[11] Naheed K, Hussain A, Ali R. Clinico-Pathological Study of Hysterectomy at Pak Red Crescent Medical and Dental College. J Islamic Int Med Coll. 2018;13(2):62–5.

[12] Jaleel R, Khan A, Soomro N. Clinico-pathological study of. Pak J Med Sci. 2009;25(4):630–34.

[13] Nyirahabimana D, Musoni E, Mbarushimana D, Rugwizangoga B. Analysis of Histopathological Lesions in Hysterectomy Specimens at Two Teaching Hospitals in Rwanda : A Two Year Review. J Gynecol Infertility. 2018;1(1):1–4.

[14] Khan S. How does histopathology correlate with clinical and operative findings in abdominal hysterectomy? J Armed Forces Med Coll. 2010;6(2):17–20.

[15] Modupeola S, Adesiyun, Agunbiade. Hysterectomies in Zaria. Eur J Gen Med. 2009;150–3. 10.29333/ejgm/82660.

[16] Daniel PD, Anupama D. To determine effectiveness of abdominal hysterectomy versus non descent vaginal hysterectomy. Int Arch Integr Med. 2017;4(10):77–86.

[17] Nilsson L, Wodlin NB, Kjölhede P. Risk factors for postoperative complications after fast-track abdominal hysterectomy. Aust N Z J Obstet Gynecol. 2012;52:113–20. 10.1111/j.1479-828X.2011.01395.x 22224504

[18] Siwatch S, Kundu R, Mohan H, Huria A. Histopathologic audit of hysterectomy specimens in a tertiary care hospital. Sri Lanka J Obstet Gynecol. 2012;34(4):155–8.

[19] Gangadharan V, Prasanthi C. Original article Hysterectomy - a clinico-pathological correlation in a rural setting. Indian J Basic Appl Med Res. 2016;5(2):8–15..

